# A Partnership Model for Improving Service Delivery in Remote Papua New Guinea: A Mixed Methods Evaluation

**DOI:** 10.15171/ijhpm.2018.50

**Published:** 2018-06-11

**Authors:** Emma Field, Dominica Abo, Louis Samiak, Mafu Vila, Georgina Dove, Alex Rosewell, Sally Nathan

**Affiliations:** ^1^Global and Tropical Health, Menzies School of Health Research, Brisbane, QLD, Australia.; ^2^Abt Associates, Brisbane, Australia.; ^3^School of Public Health and Community Medicine, University of New South Wales, Sydney, NSW, Australia.; ^4^Abt Associates, Port Moresby, Papua New Guinea.; ^5^University of Papua New Guinea, Port Moresby, Papua New Guinea.

**Keywords:** Partnership, Service Delivery, Monitoring and Evaluation, Papua New Guinea

## Abstract

**Background:** The Community Mine Continuation Agreement Middle (CMCA) and South Fly Health Program (the Health Program) is a partnership for improving health service delivery in remote Papua New Guinea (PNG). The Health Program is delivered by a private contractor working in partnership with existing health service providers to improve service delivery using existing government systems, where possible, and aligns with national policies, plans and strategies. A midline evaluation was conducted to determine changes in health service delivery since commencement of the Health Program.

**Methods:** A mixed methods evaluation was undertaken mid-way through implementation of the Health Program, including a pre/post analysis of health service delivery indicators, semi-structured interviews with health workers and assessment of health facility equipment and infrastructure.

**Results:** Improvements in many of the long-term expected outcomes of the Health Program were observed when compared to the pre-program period. The number of outpatient visits per person per year and number of outreach clinics per 1000 children under 5 years increased by 15% and 189% respectively (*P*<.001). Increases in vaccination coverage for infants aged <1 year were observed: 58 % for pentavalent 1st dose (*P*<.001) and 75% for 1st dose Sabin (*P*<.001), 30% for 3rd dose pentavalent (*P*<.001) and 26% for measles vaccination (*P*<.001). Family planning coverage remained at similar levels (increasing 5%, *P*=.095) and antenatal care coverage increased by 26% (*P*<.001). Supervised deliveries coverage declined by 32% (*P*<.001), a continuation of the pre-Program trend. The proportion of facilities with standard equipment items, transport and lighting increased. Health worker training, in particular obstetric training, was most commonly cited by health workers as leading to improved services.

**Conclusion:** Following implementation, substantial improvements in health service delivery indicators were observed in the Health Program area as compared with pre-program period and the stagnating or declining national performance. This model could be considered for similar contexts where existing health service providers require external assistance to provide basic health services to the community

## Background

One model for improving service delivery involves engaging the support of non-government or private providers to deliver defined health services.^1-5^ This contracting-out model of public-private engagement for service delivery can fill capacity gaps within governments and has been used in developing countries including Afghanistan, Ghana, Malawi, and Pakistan.^6-10^ The documented benefits from this model include improvement in the utilisation, coverage and quality of health services and more effective human resource management and procurement.^6-8,10^ However, negative outcomes associated with this model include: no improvement in quality of health services; ineffective referral; poor integration with national health programs; by-passing government process eg, for drug procurement; and concerns for sustainability.^8,10^ Evidence for the effectiveness of such models for improving health service delivery is limited, partly due to the challenge of rigorously evaluating complex service delivery programs as opposed to evaluating single interventions or packages of interventions.^11^

Practical, evidence-based delivery models for improving health services are vital. This is particularly relevant for countries like Papua New Guinea (PNG) where there has been limited improvement or in some cases declining performance in many service delivery indicators in recent years.^12,13^ A back to basics approach has been developed by the National Department of Health in PNG for implementation through the National Health Plan 2011-2020 aimed at promoting effective interventions, including the provision of the basic enablers for healthcare such as infrastructure, equipment, medical supplies, and skilled health workers as well as efforts to empower communities to address their health needs.^14^ The challenge in realising this plan remains in effective and adequately resourced implementation.^1^

Similar to contracting-out, public-private partnerships (PPP) also involve the government contracting a private organisation to deliver health services but are typically more collaborative involving shared decision making.^5^ PPP models are diverse and there are multiple definitions of what a PPP is.^15^ However, Reich^16^ describes a PPP for public health as having three points: “*First, these partnerships involve at least one private for-profit organization and at least one not-for-profit or public organization. Second, the partners have some shared objectives for the creation of social value, often for disadvantaged populations. Finally, the core partners agree to share both efforts and benefits.”* The purpose of a PPP is the provision of public goods or services, that is, services that are normally provided by a government and is delivered through a contractual agreement where assets, risks and rewards are shared between the partners.^17^ Such partnerships have been used in PNG, largely funded by the mining sector.^2^ Thomason and Rodney have suggested that PPPs in PNG can be beneficial in increasing service coverage, improving infrastructure, financial and logistical support, and capacity development.^2^ However, the nature of these partnerships and the evidence demonstrating the benefits has not described in detail.^2^

The Community Mine Continuation Agreement (CMCA) Middle and South Fly Health Program, from hereon called the Health Program, is a large complex health service program implemented in Western Province, PNG.^18^ This model is similar to a PPP and contracting-out in that a private contractor was engaged to deliver a health service delivery program in partnership with government and faith-based health service providers. However, this model differs from both contracting-out models^6-10^ and PPPs primarily in that the private organisation was contracted by a non-government organisation on behalf of the community which is funding the program. Further the private organisation works with existing health service providers to not only deliver health services but support the health service providers to improve service delivery in line with national policies, plans and strategies, using existing government systems where possible.

In 2015, a midline evaluation of the Health Program was undertaken with the objective of measuring the impact of the Health Program on service delivery 2 years into implementation. This paper describes the Health Program model and reports the changes in service delivery in the first 2 years of implementation using national data, interviews with health workers and assessments undertaken at health facilities before and mid-way through implementation.

## Methods

### Context

Western Province, bordering Indonesia, is the largest province geographically in PNG with a population of 201 351.^19^ In 2012, Western Province was ranked 17 out of the then 20 provinces of PNG in health sector performance even after adjusting for the harsh geographical constraints.^20^ A mining company, Ok Tedi Mining Limited, operates a copper mine in the North Fly District of Western Province and has made significant investment in improving health services in the district, including (1) establishing and funding the operation of a hospital in the mining town of Tabubil since the early 1980s; (2) the district-wide North Fly Health Services Development Program since 2009; and (3) the redevelopment of the district hospital since 2009.^21,22^ While there have been substantial improvements in service delivery in North Fly District,^22^ little had improved in the other two districts of the province (Middle Fly and South Fly Districts).

**Figure 1:**
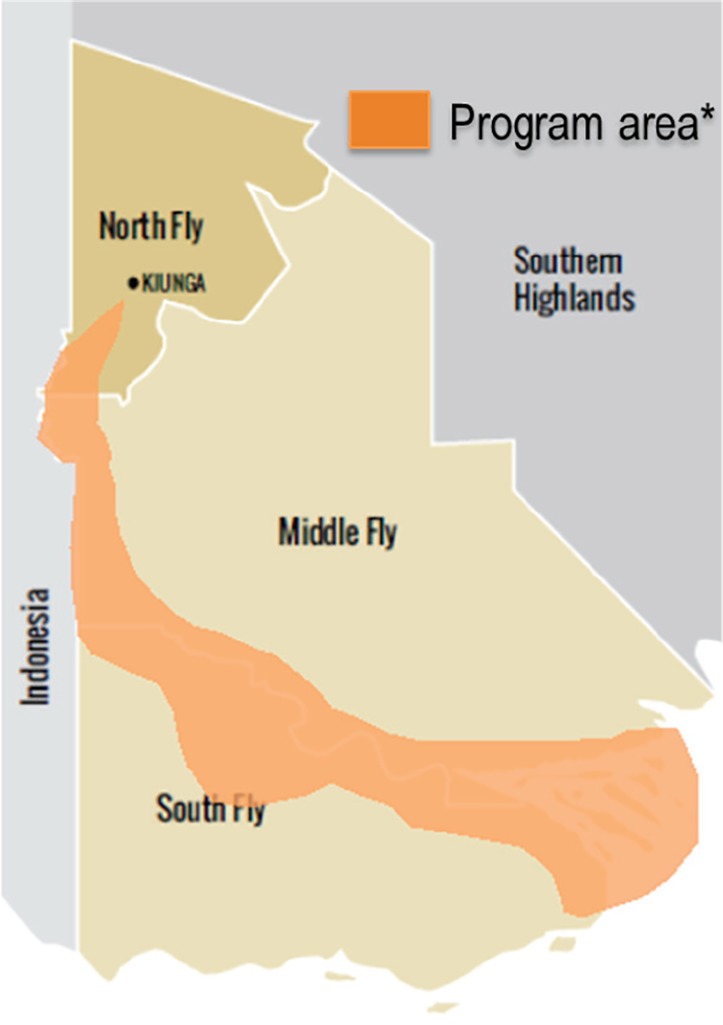
Map of Western Province, Papua New Guinea (PNG), With CMCA Middle and South Fly Health Program Area Shaded (*approximation only).

The communities impacted from the operation of the Ok Tedi Mine receive an integrated compensation and development package. The Health Program was initiated as a result of consultation with the communities in the Middle and South Fly Districts. The Health Program area covers the mine-affected communities along the Fly River from south of the town of Kiunga to the mouth of the river (Figure 1).

### CMCA Middle and South Fly Health Program Description

The CMCA Middle and South Fly Health Program, which commenced in July 2013, is a 5-year program (2013-2018) funded through Ok Tedi Development Foundation from the CMCA portion of the Western Province People’s Dividend Trust Fund. The design of the Health Program was informed by a feasibility study, commissioned by Ok Tedi Development Foundation and carried out in 2012 by Abt Associates, a health and social sector consulting company. The feasibility study involved primary data collection from health service providers, health facilities, health workers and communities and a literature review to determine the status of existing health services and priorities for improving service delivery. Extensive consultation with stakeholders including the government, provincial health office, district health offices and existing health service providers in Western Province, was undertaken to inform the program design. The feasibility study identified priorities and designed a program that was aligned with existing national and provincial health policies, strategies and plans. The resultant Health Program design was endorsed by the stakeholders. The Health Program was costed at 42 million PNG Kina (approximately US$13 million).

The model for implementing the Health Program was adapted from the North Fly Health Services Development Program as considerable progress had been made in service delivery in North Fly and many of the Health Program partners were the same.^23^ Firstly, Ok Tedi Development Foundation contracted the management and implementation of the Health Program activities to the private contractor, Abt Associates, who employed a multi-disciplinary implementation team. The implementation is guided by a Program Partnership Committee consisting of representatives from Abt Associates, Ok Tedi Development Foundation and the existing health service providers: Provincial Health Office (government), Middle Fly and South Fly District Health Services (government), Evangelical Church of PNG and Catholic Health Services. It is this committee that determines what activities will be implemented and how, aligning the activities with the National Health Plan 2011-2020 and National Health Service Standards.^14,24^ Abt Associates reports on progress quarterly to Ok Tedi Development Foundation and the Program Partnership Committee.

The implementation of the Health Program activities is supported by a team based in the province, employed by Abt Associates. The team consists of a program manager, administrative staff, logistic officers, infrastructure officers, a health information officer, a workforce training officer, village health volunteer coordinators and clinical outreach health workers. Wherever possible the Health Program team works with their counterparts in the existing health service provider organisations.

**Figure 2:**
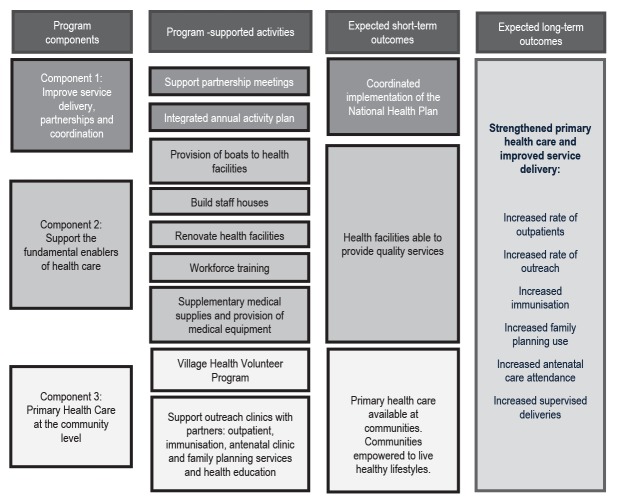
Simplified Program Logic for the CMCA Middle and South Fly Health Program. Abbreviation: CMCA, Community Mine Continuation Agreement.

The Health Program has three components: partnerships and coordination; the fundamental enablers of healthcare; and primary healthcare at the community level.^25^ Each component comprises multiple interventions (Figure 2). Component 1 aligns with the National Health Plan 2011-2020 key result areas “Improve Service Delivery” and “Strengthen Partnerships and Coordination with Stakeholders.”^14^ The Health Program activities in this component are centred on bringing stakeholders in health together to coordinate activities to improve service delivery. A key Health Program activity is facilitating quarterly partnership meetings that brings together representatives from the existing health service providers (Provincial Health Office, Middle Fly and South Fly District Health Services, Evangelical Church of PNG and Catholic Health Services) and other related non-government organisation (eg, World Vision). Prior to the Health Program some of these stakeholders did not have the opportunity to meet regularly due to being located in different areas of Western Province. These Health Program partners meet annually to develop and approve the following year’s annual workplan for the Health Program, including targets. The goals, activities and targets of the workplans are aligned with the National Health Plan and the National Health Service Standards,^14,24^ and complement the Health Program partners’ plans. The Health Program partners also meet quarterly to review progress on implementation and identify instances where adaptations to existing plans are required or priorities for annual workplans for future workplans. The progress reports utilise the Health Program monitoring and evaluation (M&E) system which includes primary data collected by team members on Health Program activities implemented for reporting progress against the annual workplans and secondary data from the National Health Information System (NHIS) for measuring outcomes.

Component 2 aligns with the key result area of “Strengthen Health Systems” of the National Health Plan 2011-2020^14^ and involves supporting the fundamental enablers of healthcare. Health Program activities include the provision of medical equipment, including medical equipment kits to meet the National Health Service Standards, vaccine fridges and health radios; renovation of health facilities including, but not limited to, provision of lighting, plumbing for running water; coordination of medical supplies ordering and distribution to health facilities though the government system; procurement of the construction of staff houses for health workers; provision of transport (dinghy and outboard motor) to health service providers for use for outreach clinics and patient transfers; and coordination and delivery of health worker training. The health worker training is either government health worker training programs, for example the Essential Obstetric Care Training, or training that is developed and delivered by the Program Team after identification of a need by the health service providers.

Component 3 focusses on the delivery of primary healthcare at the community level and aligns with the National Health Plan 2011-2020 key result areas of “Improve Child Survival,” “Improve Maternal Health,” “Reduce the Burden of Communicable Diseases,” “Promote Healthy Lifestyles,” and “Improve our Preparedness for Disease Outbreaks and Emerging Population Health Issues.”^14^ Health workers working with the Health Program Team would conduct outreach clinics with existing health service providers, addressing gaps in workforce capacity, medical supplies and transport. The health workers from the Health Program Team also boost workforce numbers and capacity at health facilities through conducting clinical attachments, where they work alongside existing health workers, at facilities in the program area. These clinical attachments are also an opportunity for existing health workers to receive brief on-the-job training from the health workers from the Health Program on a range of topics, for example health information reporting and malaria treatment. The Health Program Team also coordinate the implementation of the national Village Health Volunteer Program, whereby lay health workers in villages are trained to provide health promotion and first aid in villages and connect the community with health services.

### Study Design

A mixed methods evaluation was conducted from August to September 2015. The evaluation comprised two components: a quantitative analysis of health service delivery indicators and assessment of health facility equipment and infrastructure; and a qualitative analysis of health workers’ views on implementation.

### Study Setting

The Health Program catchment area covers 84 villages along the Fly River in Western Province. Road infrastructure is extremely limited with most travel via boat on the river or on foot from the river to villages. The Health Program covers an estimated population of 50 813, and based on the projected catchment populations from health centres and health sub-centres for 2015, about one third of the total population for the Middle and South Fly Districts. A baseline evaluation of health facilities, conducted in 2013 during program commencement, determined that there were 20 open health facilities (13 health centres/health sub-centres and seven aid posts) with a total of 38 health workers.^26^ Aid posts represent the first of seven levels of health service in PNG, provide basic health promotive and curative services and are typically staffed by one to two community health workers. Health sub-centres and health centres represent level two and three health services and provide higher level services. Complex health services are addressed the various levels of hospitals (levels 4-7).^24^

### Indicator Analysis

The indicators used for this evaluation were adopted from the *National Health Plan Monitoring and Evaluation Framework*^27^ These indicators relate to the long-term expected outcomes in the program logic (Figure 1) and were calculated for the Health Program area. Specifically, the outcome “increased rate of outpatients” is measured through the indicator “outpatients per person per year,” calculated by dividing the total number of outpatients seen by facilities in the Health Program area by the catchment population in the Health Program area. The outcome “increased rate of outpatients” is measured by the indicator “rate of outreach clinics per 1000 children <5 years of age,” calculated by dividing the number of outreach clinics held in the Health Program area by the number of children <5 years of age, then multiplied by 1000. “Increased immunisation” was measured through vaccination coverage of children <1 year of age for the third dose of pentavalent vaccine (vaccine for diphtheria, tetanus, whooping cough, hepatitis B and Haemophilus influenza type b) and the 9-11 month dose of measles vaccine. Vaccine coverage (%) was calculated by dividing the number of the specified doses by the number of children <1 year of age, multiplied by 100. “Increase family planning use” was measured by the indicator “Couple Years Protection per 1000 women of reproductive age (15-44 years),” calculated by the estimated months protection provided by each contraceptive method, multiplied by the number of each contraceptive distributed during that year, divided by the number of women of reproductive age, multiplied by 1000. “Increased antenatal care” was measured through the indicator “proportion (%) of pregnant women who have received at least one antenatal care visit,” calculated by the number of first antenatal care visits divided by the estimated number of pregnant women, multiplied by 100. “Increased supervised deliveries” was measured through the indicator “proportion of women who had a supervised delivery,” calculated by the number of deliveries supervised by either a health worker or village birth attendant divided by the estimated number of births, multiplied by 100.

In addition to the national indicators, additional indicators on vaccination were included in the analysis to measure the impact of the immunisation related activities (eg, installation of vaccine fridges, vaccinations administered through Health Program supported outreach clinics). These indicators included: pentavalent first dose coverage for children <1 year of age; sabin first and third dose coverage for children <1 year of age; and number of measles, sabin and pentavelnt vaccines administered for the >1 year age. Coverage for the >1 year group could not be calculated as there is no upper age limit for the group. The number of child attendances per 1000 children <5 years of age was also included as an indicator to further measure the change in service delivery at the health facilities.

All numerators and denominators for indicators were taken from the annual NHIS reports for Western Province for 2010-2015. In 2015, there were 22 health facilities in the Health Program catchment area: 14 aid posts and eight health centres/sub-health centres. The aid posts report NHIS data to the closest of the eight Health Centres/Health Sub-Centres and these eight facilities report NHIS data through to the provincial level. Completeness for reporting ranged from 82% to 100% over the 6 years in the Health Program area. Data were adjusted for missing reports by dividing the annual totals by the reporting rate. To calculate the indicators, the totals from the eight reporting facilities were combined and divided by the combined catchment population. For Samari Health Sub-Centre, data for 2010 and 2011 were adjusted for missing reports but not for 2012-2015 as the facility closed in 2012. Samari Health Sub-Centre reopened in 2015 but only periodically as only one health worker was present. However, data from outreach clinics conducted in the catchment area of Samari Health Sub-Centre were reported under Samari Health Sub-Centre in the NHIS for the duration of 2012-2015 and have been included without adjustment. A sensitivity analysis was performed comparing the results excluding and including Samari Health Sub-Centre, with no significant difference observed.

Statistical analysis of the change in the indictors between 2010-2012 and 2014-2015 was undertaken by calculating the incidence rate ratio for all indicators involving rates or % coverage. For the indicators involving total numbers only (number of pentavalent, measles and sabin vaccine administered for the <1 year age group) *t* tests were performed. Analyses were performed in Stata 15. Where available, the reported national level performance for indicators was included for comparison using the figures from the Sector Performance Annual Reviews.^12,20^

### Health Facility Assessment

A purposive sample of ten health facilities were visited during the midline data collection period from August to September 2015 to ensure representation from each of the geographic regions, the three levels of health facilities (aid posts, health sub-centres and health centres) and the three health service provider organisations (District Health Services, Evangelical Church of PNG and Catholic Health Services). The medical equipment available was assessed at nine of these health facilities through visual inspection. The data from the midline evaluation was compared to data available for the same facilities from the baseline evaluation conducted in 17 of the 20 open health facilities at the commencement of the Health Program.^28^ In addition, data from the Health Program M&E system on health facility equipment and infrastructure were used to assess changes.^29^

### Health Worker Interviews

All available health workers at the ten health facilities visited during the midline data collection from August to September 2015 were invited to participate in a semi-structured face-to-face interview. The interview topics included changes since the Health Program commenced, and barriers and enablers to providing quality health services. The key informant interviews were carried out either in English or Tok Pisin. The audio was recorded on a digital recorder and was transcribed. If the audio was in Tok Pisin, it was translated into English and transcribed. The transcriptions were imported into NVivo (QSR International, USA). The qualitative analysis was conducted by one author (EF). Firstly interviews were coded with their location and then the response coded to each question. Interview transcripts were then coded for themes and concepts corresponding to the domains included in the interview guide: perceived changes in health service delivery related to the Health Program and perceived barriers and enablers for health service provision. Thirdly inductive thematic analysis was used for coding the specific changes, barriers and enablers.^30^

## Results

### Health Facility Infrastructure and Equipment

The number of health facilities open increased from 20 to 21, with one facility reopening after receiving renovations through the Health Program during the 2-year program period. Assessments of availability of equipment were completed at nine of the ten health facilities visited during the evaluation, as one facility visited had just reopened at the same time as the midline evaluation. A direct comparison of the nine facilities from the midline to the baseline is not presented as only eight of these facilities had baseline data. However, analysis of this subset eight of facilities produces a similar result to that presented in Table 1 (data not shown). Availability of transport, equipment and infrastructure at health facilities improved in the first 2 years of the Health Program (Table 1).

**Table 1:** Health Facilities Assessment at Baseline and Midline Evaluation

**Health Facility Assessment Criteria**	**Baseline (2013)** ^ a,b ^	**Midline (2015)** ^b^
Health facilities open	20	21^c^
Health centres/health sub-centres open	7	7^c^
Aid posts open	13	14^c^
% of facilities with transport available (dinghy and outboard motor)	0% (0/17)	67% (14/21)^c^
% of facilities with working vaccine refrigerator	30% (6/17)	76% (16/21)^c^
% of facilities with working health radio	47% (8/17)	100% (21/21)^c^
% of facilities with lighting	24% (4/17)	100% (21/21)^c^
% facilities with running water	12% (2/17)	38% (8/21)^c^
Aggregate number of staff houses^e^	18	22^c^
% of facilities with the following medical equipment		
Adult scales	88% (15/17)	100% (9/9)^d^
Infant (hanging) scales	71% (12/17)	89% (8/9)^d^
Steriliser, pressure cooker	0% (0/17)	67% (6/9)^d^
Primus stove	12% (2/17)	89% (8/9)^d^
Auroscope	41% (7/17)	78% (7/9)^d^
Foot/electric nebuliser	24% (4/17)	56% (5/9)^d^
Aneroid sphygmomanometer	59% (10/17)	100% (9/9)^d^
Bell stethoscope	65% (11/17)	100% (9/9)^d^
Clinical oral thermometer	88% (15/17)	100% (9/9)^d^

^a^ Data collected through direct observation during the baseline evaluation where 17 of the 20 open health facilities were visited in 2013. ^b^ Figures in parenthesis represent the numerator and denominator. c^c^ Data collected through the Program Monitoring and Evaluation System for the 21 open health facilities in 2015. ^d^ Data collected through direct observation during midline evaluation at nine of the 21 open health facilities in 2015. ^e^ Staff numbers did not change substantially during this period.

### Health Service Delivery

There were substantial increases in outpatient visits, child attendances, outreach clinics, vaccination and antenatal care in the Program period compared to the pre-Program period (Table 2).

**Table 2:** Change in Service Delivery Indicators Prior to (2010-2012) and After (2014-2015) Health Program Commencement in the Health Program Area and National Level

**Indicators**	**Pre-program Period**	**Program Period**	**% Change 2010/2012-2014/2015** ^a^	**IRR (95% CI)**	***P*** ** Value**
Health service utilisation					
Outpatient visits per person per year (Program)	1.3	1.5	**15.3**	**1.2 (1.1-1.2)**	**<.001**
Outpatient visits per person per year (National)	1.4	1.2	-11.6	-	-
Child attendances per 1000 children < 5 years (Program)	0.7	1.3	**87.7**	**1.9 (1.8-1.9)**	**<.001**
Outreach clinics per 1000 children <5 years (Program)^c^	9.3	27.0	**189.3**	**2.9 (2.5-3.5)**	**<.001**
Outreach clinics per 1000 children <5 years (National)	38.0	38.0	0.0	‏-	‏-
Vaccination <1 year of age					
Pentavalent 1st dose coverage (%) (Program)	43.7	69.0	**58.0**	**1.6 (1.5-1.7)**	**<.001**
Pentavalent 3rd dose coverage (%) (Program)	18.0	23.5	**30.6**	**1.3 (1.2-1.4)**	**<.001**
Pentavalent 3rd dose coverage (%) (National)	50.3	57.5	14.2	-	-
Measles 9-11 months coverage (%) (Program)	28.7	36.0	**25.6**	**1.2 (1.1-1.3)**	**<.001**
Measles 9-11 months coverage (%) (National)	49.0	51.0	4.1	-	-
Sabin 1st dose coverage (%) (Program)	46.3	81.0	**74.9**	**1.6 (1.5-1.7)**	**<.001**
Sabin 3rd dose coverage (%) (Program)	32.0	36.0	12.3	1.0 (0.9-1.1)	.740
Vaccination >1 year of age					
Pentavalent (#) (Program)	511	1630	**219.0**	-	**.008**
Sabin (#) (Program)	943	1940	**105.7**	-	**.039**
Measles (#) (Program)	934	3779^b^	304.6^b^	-	.102
Maternal and reproductive health					
Couple years protection per 1000 WRA(Program)	80.7	85.0	5.2	1.1 (1.0-1.1)	.095
Couple years protection per 1000 WRA(National)	79.0	64.5	-18.2	-	-
ANC 1st visit coverage (%) (Program)	31.0	39.0	**25.8**	**1.3 (1.2-1.4)**	**<.001**
ANC 1st visit coverage (%) (National)	64.3	65.0	1.0	-	-
Supervised deliveries coverage (%) (Program)	17.7	12.0	**-32.1**	**0.7 (0.6-0.7)**	**<.001**
Supervised deliveries coverage (%) (National)	41.3	40.5	-2.0	-	-

Abbreviations: IRR, incidence rate ratio; ANC, Antenatal care; WRA, women of reproductive age (15-44 years of age). ^a^ Percent change calculated from mean post program commencement years (2014-2015) divided by mean pre-program commencement years (2010-2012). Data for 2013 were excluded as the program commenced in July 2013. ^b^ In 2014 there was supplementary immunisation activity targeting immunisation of children aged from 6 months to adults of 20 years of age in response to a measles outbreak which likely inflates these figures. ^c^ Anomalous data from Wasua Sub-Health Centre excluded for 2010.

#### Health Service Utilisation

There was strong evidence (*P *< .001) that outpatients and outreach clinics increased in the Program period compared to the pre-Program period. There was a 15% and 189% increase in outpatient visits per person per year and outreach clinics per 1000 children <5 years of age respectively in the Program area. For comparison, national outpatient visits per person per year declined by 12% and there was no change in outreach clinics per 1000 children <5 years of age.

#### Vaccination

Vaccination coverage for children <1 year of age for pentavalent 3rd dose and the measles 9-11 month dose increased by 30% and 26% respectively (*P *< .001), while at the National level there was a respective 14% and 4% increase. However, reported overall coverage pentavalent third dose and the measles 9-11 month dose for children <1 year was low in the Health Program area compared to National level at 30% and 34% respectively. In contrast, first dose coverage for pentavalent and Sabin vaccination had higher increases in coverage (58% and 75% respectively, *P *< .001) and higher resultant coverage in the Program period (71% and 80% respectively). Furthermore, there were large increases in the number of vaccinations given to the >1 year age group ranging from 106% for Sabin vaccination (*P *= .039) to 305% for measles vaccination (*P *= .102). The large increase in the number of measles vaccinations administered coincided with a supplementary immunisation activity targeting immunisation of children aged from six months to adults of 20 years of age in response to a measles outbreak.

#### Maternal and Reproductive Health

Family planning coverage (couple years protection per 1000 women of reproductive age) increased by 5% in the Health Program area although the strength of evidence for this increase was lower than other indicators (*P *= .095). This contrasts an 18% decrease in couple years protection per 1000 women of reproductive age at the national level. Antenatal care 1st visit coverage increased by 26% in the Program area (*P *< .001) and by just 1% nationally. Supervised deliveries coverage in the Health Program area significantly declined 32% (*P *< .001), while supervised deliveries declined by 2% nationally.

### Health Worker Perspectives of the Health Program

Twenty-two health workers were interviewed comprising between one and five health workers at the ten selected health facilities, representing 58% of all health workers and 45% of health facilities in the Health Program area.

#### Perceived Changes in Health Service Delivery Due to the Health Program

The main changes the health workers noted since the commencement of the Health Program were the improvements in health facility infrastructure, equipment, access to training, transport (dinghies and outboard motors), facility lighting and running water.

“*Yeah I have seen some changes [to the health facility]. The [facility] look like a new [facility] where it was a condemned building [before] and we had a lot of rusted instruments and what was inside was not looking good. But since they came and helped, did the maintenance, we are happy about the appearance of the [facility] now.”*

“*I find it hard to treat patients in the night. But now I’m very happy that I’ve got good lights so that I can help my own patient coming into the aid post. Another great change is that I have a very good radio for communication in case of emergencies.”*

When asked what had led to improved services at the health facility, the most common response was training. Training for health workers included officer-in-charge training, essential obstetric care (EOC) training, basic management training for rural health workforce, provider initiated counselling and testing for HIV. The training that health workers cited most commonly as resulting in changed practices was the EOC training. For example, one health worker changed the availability of antenatal care services:

*Before we had certain days to attend to antenatal matters, but when I went for the EOC [essential obstetric care] course I was taught to attend to the mother when she comes at any time … We had certain days [for antenatal care] but now they come in whichever time we give them for their next date of visit.*

In contrast to the quantitative data indicating a decline in supervised deliveries coverage in the Health Program area, several health workers cited that there were now more supervised deliveries as a result of the EOC training. Supervised deliveries were also reported to increase after renovations to the health facility as reported by one health worker:

“*So the changes like building this new labour ward. So we have seen the improvements nowadays. All the ladies coming in and delivering in these ward facilities.”*

However, a lack of a dedicated space within other health facilities for deliveries was noted by some health workers as a barrier to supervised deliveries in the health facility:

*At the moment we don’t have facilities for the deliveries that we can do in the facility. We go out to the bush or to their homes and help women deliver.*

#### Perceived Ongoing Challenges of Health Service Delivery

More broadly, health workers cited a range of barriers to providing effective health services including a lack of basic supplies (fuel for transport, medical supplies), lack of supervision, lack of community support and cultural barriers that prevented people from accessing services (female health worker treating males, lack of male participation in maternal and reproductive health, and a perception that different treatment provided by church run services for people from different religions).

While health workers noted that the provision of a dinghy and outboard motor through the Health Program was helpful for transfer of patients to higher level health facilities, the lack of access to a regular supply of fuel was an issue. Health workers expected the fuel to be provided either through the Health Program or their health service provider organisation.

When probed further on the topic of supervision, health workers cited that they rarely received supervisory visits from their supervisors at their health service provider organisation. When supervisory visits did occur, many health workers were not satisfied with the supervision they received as they did not think they received adequate advice or training.

*“I really want supervision to see whether I’m improving in my job or I’m still at the same level… If only they would come and see my workload or whatever things that they will maybe appreciate me for my job. I will still change my attitude or bring my standard up a bit myself”* (Health Worker).

## Discussion

After 2 years of implementation, the Health Program has contributed to an improvement in health facility infrastructure and equipment and to an improvement in health service delivery as demonstrated through increases in the outreach (189% increase), outpatients (15%), immunisation (26% for measles and 31% for pentavalent) and antenatal care (26%). Health workers noted improvements in equipment, access to training, transport, facility lighting and running water and cited training as the intervention that resulted in changed practices. These improvements in service delivery in the Health Program area have occurred in the context of stagnating or declining national performance in health indicators.

The mining sector has contributed to health service delivery through single disease programs (eg, HIV) to large complex health service programs.^2,31-33^ This study offers support for the effectiveness of a mining company’s contribution to the health sector, of which evidence is limited, particularly for PNG.^34^ Another program in PNG, the North Fly Health Services Development Program in the North Fly District of Western Province, works in partnership with the same health service providers, is funded directly by Ok Tedi Mining Limited and is implemented by private contractor Abt Associates. Within the first two years of the Health Program implementation there were increases in outreach, immunisation, and first antenatal care visits coverage in North Fly, similar to those we presented in this study for the CMCA Middle and South Fly Health Program.^21^

One difference between these two programs is that there were substantial increases in supervised deliveries observed for the North Fly Health Services Development Program, and a decline observed for this Health Program. The decline in supervised deliveries occurred despite an increase in antenatal care visit coverage, which is a predictor of women having a supervised delivery.^35^ The North Fly Health Services Delivery Program differs from this Health Program in that it supports the whole district with the exception of two hospitals that have separate initiatives to improve services, also funded through Ok Tedi Mining Limited.^22,36^ Women may prefer to deliver at these higher level health facilities and this may partly explain the higher supervised delivery coverage observed after the commencement of the North Fly Health Services Development Program compared to this Health Program. The health worker interviews suggested improvements in antenatal care and supervised deliveries following the Essential Obstetric Care training although only improvements in antenatal care were seen in the indicator analysis. The health worker interviews revealed that the lack of a suitable space within the facility for women to deliver, which may have contributed to fewer births in the facility and more births supervised in the villages. Village births that are supervised by a trained health worker are considered a supervised delivery but may be underreported compared to facility births. Further investigation is required to understand the low supervised delivery coverage in the Health Program area.

The largest improvements noted in the evaluation were for immunisation, particularly for the greater than one-year age group. The improvements in immunisation were likely assisted by the installation of vaccine refrigerators through the Health Program. However, immunisation coverage for third does pentavalent and Sabin and the measles 9-11 month dose were low. Provision of vaccination for three doses of pentavalent or Sabin vaccine requires three interactions with health workers, either through outreach clinics or at health facilities, in the first year of life, assuming availability of vaccine at the time of interaction. As documented through the qualitative data, the lack of fuel for transport for health workers, which would be used for outreach clinics where vaccinations are provided, remains a challenge in this setting. Poor transport also been documented to be a contributor to lower coverage in rural and hard to reach locations.^37^

Few international studies have quantified the changes in service delivery indicators following the introduction of contracting for health service delivery. The implementation of the Basic Packages of Health Services in Afghanistan through a model where non-governmental organizations (NGOs) were contracted to provide services demonstrated similarly large increases in service delivery indicators in the early years of implementation followed by much smaller improvements in the following years.^7^ This phenomenon was observed for the Health Program in this study large improvements were seen in the first year of full implementation (2014) compared to 2012, followed by smaller changes between 2014 and 2015 (data not shown). In Malawi, the introduction of contracting a faith-based health service provider was associated with large increases in child attendances and deliveries, although this coincided with the removal of user fees which could also have affected utilisation.^8^

This evaluation describes a program for service delivery in a remote, resource limited setting delivered through a partnership between a private contractor, non-government organisations and government. While the program differs from a PPP in that the contract is between a NGO (Ok Tedi Development Foundation) and a private contractor rather than between the government and private contractor, there are elements that are similar to PPPs in that the program delivered services to the public in partnership with government and faith-based organisations maximising the use of staff and resources of all partners through shared planning and implementation. The vast majority of literature on health PPPs to date is focussed on the developed setting, eg, Europe, and on infrastructure PPPs where by the contractor builds and maintains the health service and may also operate the health service.^38^ PPPs are seen as an alternative funding mechanism for healthcare capital where government resources are limited.^39^

The Health Program brought in additional resources but also promoted more efficient use of resources in the Health Program area for all health service providers. The feasibility study and baseline evaluation provided a detailed assessment of the status of health services in the Health Program area and the gaps that needed to be addressed. Activities and resources were better coordinated between health service providers and the Health Program to achieve the shared goals of implementation the National Health Plan 2011-2020. Regular partnership meetings for annual planning and quarterly reviews of progress assisted with creating shared understanding of health service delivery in the area. Both coordination and mutual knowledge have been identified as positively impacting partnerships between the public and private sector.^40^ Further Kivleniece and Quelin propose that an integrative mode of governance, as used for the Health Program, creates value through complementary use of the public and private sector skills and resources.^41^

There are limitations to the evaluation. Firstly, there are likely to be some issues with the completeness and quality of the NHIS and population data.^42^ The data for 2014 and 2015 was quality checked, through review of monthly reports and following up with health facilities if data were missing or appears anomalous, but it was not possible to do so for the earlier years due to the absence of the hard copy monthly reports. The annual data for 2010-2013 were reviewed for anomalies and only one figure appeared anomalous – the number of outreach clinics for Wasua Sub-Health Centre for 2010 was unusually high without any other explanation. This was addressed this issue by removing the data for Wasua Sub-Health Centre from the calculation of the indicator for 2010. Any other inaccuracies with the data that have not been detected may mean the exact magnitude of the change in indicators may be inaccurate; however, the overall improvement of services was largely correlated between the NHIS data and qualitative data from the health workers. Secondly, the evaluation was a before-after design and did not include controls. While the changes in service delivery in the Health Program area may have been due to external factors not documented in the evaluation, this is unlikely as the main source of external support for the health facilities was the Health Program and similar increases in indicators were not seen at the national level. Thirdly, as the nature of the Health Program involves multiple interventions it is not possible to determine which were essential in reaching the outcomes of improved service delivery. Nonetheless, the strength of the evaluation is the use of mixed methods including analysis of indicators, interviews with health workers and facility visits to triangulate results.

The Health Program has now entered the transition phase, where resources will gradually taper as existing health service providers’ capacity improves to foster sustainable service delivery. The evaluation has identified of areas for improvement to be addressed through future implementation of the Health Program, for supervised deliveries and supervision of health workers. Following lessons from successful transitions of donor-funded program to country ownership elsewhere,^43^ the Health Program team has heavily involved existing health service providers in transition planning and identified the gaps to be addressed before the end of the Health Program in 2018.

## Conclusion

This evaluation has demonstrated that a program delivered through a partnership between government, and private and faith-based organisations has contributed to progress in improving service delivery in remote PNG through the use of funds provided by a mining company to the community. Elements of the Health Program model included: a feasibility study to inform the Health Program design; governance through the funding agency and existing health service providers; alignment of workplans with national plans and standards; joint annual planning with existing health service providers; and a focus on addressing gaps in health service delivery. This model could be considered for similar contexts in PNG and in other countries where existing health service providers require external assistance to provide basic services to the community.

## Acknowledgements

The authors would like to acknowledge the health workers who participated in this evaluation.

## Ethical issues

All key informant participants provided written informed consent. All transcripts of interviews were de-identified. The midline evaluation was approved by the PNG Medical Research Advisory Committee (MRAC No. 15.08) and University of New South Wales Human Research Ethics Committee (HC15466).

## Competing interests

EF, GD, and MV were employees at Abt Associates at the time of the evaluation and part of the CMCA Middle and South Fly Health Program team. LS and DA were engaged as consultants through Abt Associates for the evaluation. The evaluation was funded as part of the core activities for CMCA Middle and South Fly Health Program. Abt Associates designed and implements the CMCA Middle and South Fly Health Program and is committed to acting on the findings of the evaluation, both positive and negative, to improve the program. SN and AR declare no conflicts of interest.

## Authors’ contributions

All authors contributed to the study design. DA, LS, and MV undertook data collection. EF undertook the data analysis. All authors contributed to the interpretation of the results. EF led the writing of the manuscript with input from all other authors. All authors reviewed and approved the final manuscript.

## Authors’ affiliations

^1^Global and Tropical Health, Menzies School of Health Research, Brisbane, QLD, Australia. ^2^Abt Associates, Brisbane, Australia. ^3^School of Public Health and Community Medicine, University of New South Wales, Sydney, NSW, Australia.^4^Abt Associates, Port Moresby, Papua New Guinea. ^5^University of Papua New Guinea, Port Moresby, Papua New Guinea.

## Key messages

**Implications for policy makers**

- Health service delivery in Papua New Guinea (PNG) has been stagnating in recent years. New models for improving service delivery are required.
- This study describes a partnership in remote PNG where a private contractor works with existing government and faith-based health service providers to improve service delivery.
- The evaluation demonstrated the Health Program contributed to improvements in service delivery, including outpatient visits, outreach clinics, immunisations and antenatal care.

**Implications for the public**

The study describes an evaluation of the Community Mine Continuation Agreement (CMCA) Middle and South Fly Health Program (the Health Program) in remote Papua New Guinea (PNG). The Health Program was delivered through a partnership, where a private contractor worked with existing health service providers to improve service delivery using existing government systems, where possible, and aligned activities with national policies, plans and strategies. The implementation of the Health Program was associated with an improvement in health service delivery, including increased outpatient visits, outreach clinics, immunisations and antenatal care. This model of health service delivery could be considered for similar contexts where existing health service providers require external assistance to provide basic services to the community.
